# Harnessing the power of native biocontrol agents against wilt disease of Pigeonpea incited by *Fusarium udum*

**DOI:** 10.1038/s41598-024-60039-0

**Published:** 2024-05-31

**Authors:** B. Deepak Reddy, Birendra Kumar, Sangita Sahni, G. Yashaswini, Somala Karthik, M. S. Sai Reddy, Rajeev Kumar, U. Mukherjee, K. Sai Krishna

**Affiliations:** 1 Department of Plant Pathology, Dr. Rajendra Prasad Central Agricultural University, Pusa, Bihar India; 2 Department of Entomology, Dr. Rajendra Prasad Central Agricultural University, Pusa, Bihar India; 3 Department of Agricultural Biotechnology and Molecular Biology, Dr. Rajendra Prasad, Central Agricultural University, Pusa, Bihar India; 4 Department of Basic Sciences and Languages, Dr. Rajendra Prasad Central Agricultural University, Pusa, Bihar India

**Keywords:** Defence enzymes, Field trials, PGPR, *P*. *aeruginosa*, *Trichoderma*, Microbiology, Plant sciences

## Abstract

*Fusarium* wilt, caused by (*Fusarium udum* Butler), is a significant threat to pigeonpea crops worldwide, leading to substantial yield losses. Traditional approaches like fungicides and resistant cultivars are not practical due to the persistent and evolving nature of the pathogen. Therefore, native biocontrol agents are considered to be more sustainable solution, as they adapt well to local soil and climatic conditions. In this study, five isolates of *F*. *udum* infecting pigeonpea were isolated from various cultivars and characterized morphologically and molecularly. The isolate from the ICP 8858 cultivar displayed the highest virulence of 90%. Besides, 100 endophytic bacteria, 100 rhizosphere bacteria and three *Trichoderma* spp. were isolated and tested against *F*. *udum* isolated from ICP 8858 under in vitro conditions. Out of the 200 bacteria tested, nine showed highest inhibition, including Rb-4 (*Bacillus* sp.), Rb-11 (*B*. *subtilis*), Rb-14 (*B*. *megaterium*), Rb-18 (*B*. *subtilis*), Rb-19 (*B*. *velezensis*), Eb-8 (*Bacillus* sp.), Eb-11 (*B*. *subtilis*), Eb-13 (*P*. *aeruginosa*), and Eb-21 (*P*. *aeruginosa*). Similarly, *Trichoderma* spp. were identified as *T*. *harzianum*, *T*. *asperellum* and *Trichoderma* sp. Notably, Rb-18 (*B*. *subtilis*) and Eb-21 (*P*. *aeruginosa*) exhibited promising characteristics such as the production of hydrogen cyanide (HCN), cellulase, siderophores, ammonia and nutrient solubilization. Furthermore, treating pigeonpea seedlings with these beneficial microorganisms led to increased levels of key enzymes (POD, PPO, and PAL) associated with resistance to *Fusarium* wilt, compared to untreated controls. In field trials conducted for four seasons, the application of these potential biocontrol agents as seed treatments on the susceptible ICP2376 cultivar led to the lowest disease incidence. Specifically, treatments T2 (33.33) (*P*. *aeruginosa*) and T3 (35.41) (*T*. *harzianium*) exhibited the lowest disease incidence, followed by T6 (36.5) (Carbendizim), T1 (36.66) (*B*. *subtilis)*, T4 (52.91) (*T*. *asperellum*) and T5 (53.33) (*Trichoderma* sp.). Results of this study revealed that, *P*. *aeruginosa* (Eb-21), *B*. *subtilis* (Rb-18) and *T. harzianum* can be used for plant growth promotion and management of *Fusarium* wilt of pigeonpea.

## Introduction

Pigeonpea (*Cajanus cajan* (L.) Millsp.) holds a crucial position as a significant legume pulse crop globally, particularly in Southern and Eastern Africa, Asia, and South America, where it plays a major role in supporting the livelihoods of subsistence farmers^1^. In India pigeonpea cultivated in 45 Lha, with annual production of 42 Lt and contributing nearly 90% of world’ acreage and production^2^. Despite its importance, the crop faces considerable challenges, especially from biotic stresses, with *Fusarium* wilt caused by *Fusarium udum* being a major threat and causing substantial yield losses^3,4^. *Fusarium* wilt exhibits patchy symptoms during both seedling and adult stages, with yield losses varying depending on the stage of infection, ranging from 100% at the prepodding stage to 67% at pre-harvest and 30% at maturity. In severe cases, grain yield losses can reach up to 100% ^5–7^. The pathogenic *F*. *udum* resides in the soil, entering plants through root tips and disrupting water and mineral transport in vascular bundles. Initial symptoms include interveinal chlorosis and reduced leaf turgidity, progressing to distinctive features like a purple band spreading upward from the stem base and longitudinally split open stems displaying brown discoloration of vascular tissues^5,8,9^. Current management strategies primarily rely on chemical fungicides, but their effectiveness is limited and impractical for established crops due to pathogens soil borne nature. Concerns about fungicide resistant pathogens underscore the urgent need for sustainable and ecofriendly alternatives. A promising approach involves utilizing beneficial microbes as a substitute or complement to chemical management^10,11^. Beneficial microbes have the potential to combat pathogens and promote plant growth, offering valuable contributions to disease control and increased crop yields. Additionally, the success of biological control agents is often higher when they originate from the local environment, such as rhizosphere microbes and endophytes, compared to foreign microorganisms. Native microorganisms are well adapted to specific local conditions, including climate, soil characteristics, and soil microbiota. Notable examples of beneficial rhizosphere and endophytic microbes include *Bacillus* spp., *Pseudomonas* spp., and *Trichoderma* spp. In the rhizosphere, *Trichoderma* spp. act as effective biocontrol agents against soil borne pathogens, reducing *F*. *udum* populations and mitigating pigeonpea wilt through mechanisms like mycoparasitism, lytic enzyme production, nutrient competition, and the secretion of pathogen fighting secondary metabolites^12–14^. These interactions also impact plant biochemistry, leading to increased lignin deposition, higher phenol levels, and changes in enzyme activity in response to pathogen attacks^15^.

In both the rhizosphere and as endophytic bacteria, *Bacillus* spp. and *Pseudomonas* spp. employ various strategies to combat plant diseases, including antibiosis, lytic enzymes, resource competition, extracellular proteins, antifungal antibiotics, lipopeptides, siderophores, and hydrogen cyanide (HCN) production^16^. Additionally, these bacteria enhance nutrient availability to plants by mobilizing essential minerals such as phosphorus, potassium, and zinc through the production of organic acids^17–19^. Furthermore, *Bacillus* spp. and *Pseudomonas* spp. utilize induced systemic resistance (ISR) as a crucial mechanism to protect plants from specific diseases^20,21^. ISR involves altering cell wall structure and producing phytoalexin rich glycoproteins, pathogenesis related (PR) proteins, and hydroxyproline rich glycoproteins^22^ Plant growth promoting rhizobacteria (PGPR) strains contribute by generating antioxidant enzymes such as peroxidase (POD), phenylalanine ammonia lyase (PAL), and polyphenol oxidase (PPO), which serve as triggers for ISR in plants ^22^. Peroxidase is essential for processes like lignification, suberization, and the synthesis of phenols and glycoproteins, strengthening the plant cell wall and preventing fungal invasion^23–25^. Phenylalanine ammonia lyase, the initial enzyme in the phenylpropanoid pathway, is involved in the production of phytoalexins, phenols, and lignin. *Bacillus* spp. and *Pseudomonas* spp. enhance chitinase, PAL, PPO, Superoxide dismutase, and β-1,3-glucanase activity while inhibiting the production of polymethyl galacturonase by *F*. *udum* in pigeonpea^26^.

In the context of our study, we highlight the importance of utilizing native biocontrol agents, both fungal and bacterial, isolated from the rhizosphere and within plant tissues. These native bioagents offer distinct advantages, as they are well adapted to local soil and climatic conditions. Fertile alluvial soils with high organic matter in Bihar soils favour the growth of bioagents that can effectively manage wilt diseases.

## Materials and methods

### Seed material

Pigeonpea seeds of different cultivars were obtained under AICRP on (All India Coordinated Research Project) Pigeonpea wilt programme from IIPR (Indian Institute of Pulse Research) Khanpur.

### Collection, isolation and characterization of the pathogen

Pigeonpea plants exhibiting typical wilt symptoms were collected from highly susceptible cultivars (ICP2376 and BAHAR), moderately resistant cultivar (ICP 8862) and resistant cultivars (ICP8858 and ICP9174) at the AICRP on Pigeonpea wilt disease sick plot located at Tirhut College of Agriculture, Dholi (25° 59′ 41.9″ N latitude and 85° 35′ 43.3″ E longitude). Stem segments showing vascular discoloration were collected, surface sterilized [(70% alcohol (30 s), 1% sodium hypochlorite (30 s) and sterile distilled water (3 × 60 s)] inoculated to Potato Dextrose Agar (PDA) medium and then incubated at 25 ± 2 °C for 72 h^27^. Colonies exhibiting growth with characteristic *Fusarium* morphology were selected, subcultured, and grown on PDA medium following the methods outlined by^28,29^. Cultural characteristics, such as growth rate, growth pattern, mycelial color, pigmentation, radial growth, and zonation, were recorded after an 8 day incubation period. Microconidia and macroconidia morphology were observed after 8 and 15 days of incubation, respectively.

### Pathogenicity test

To study the pathogenicity and identity of the isolated fungus as *Fusarium*, Koch's postulates were conducted on the susceptible Pigeonpea cultivar ICP2376. Purified *Fusarium* cultures were grown in 250 mL conical flasks containing 100 g of sorghum grains, which were autoclaved at 121.8 °C under 15 lb pressure for 15 min. Following inoculation, the cultures were incubated for 15 days. The prepared inoculum was then mixed with sterilized sandy loamy soil at a 1:4 ratio (pathogen to soil, w/w) and placed in 15 cm diameter plastic pots. Pigeonpea seeds were subjected to surface sterilization with a sodium hypochlorite solution for 2 min, followed by three rinses with sterile distilled water. Each plastic pot accommodated 10 seedlings, with a group of pots without the pathogen serving as a control^30^. Wilt symptoms were observed and documented 45 days after sowing.

Percent Disease Incidence (PDI) was calculated by the formula

$${\text{PDI}}= \frac{\mathrm{No\,of\,wilted\,plants}}{\mathrm{Total\,no\,of\,plants}}\times 100$$

Similarly, the Translation Elongation Factor 1-α gene (TEF1α) and Internal Transcribed Spacer region gene (ITS) of the *Fusarium* isolates were amplified, and the sequences were submitted to NCBI GenBank for further analysis and documentation.

### Collection and isolation of biocontrol agents

Ten rhizosphere soil samples and plant samples were collected from the Samastipur and Muzaffarpur districts in Bihar, characterized by temperatures ranging from 20 to 40 °C and an annual average temperature of approximately 26 °C (Supplementary Fig. 1).

To isolate rhizobacteria and *Trichoderma* spp., 10 g of rhizosphere soil was mixed with 90 mL of sterile distilled water and serially diluted up to 10^–6^^31^. From 10^–4^ to 10^–6^ dilutions, an aliquot of 0.1 mL soil microbial suspensions were evenly spread over Nutrient Agar, King’s B, and *Trichoderma*-specific medium (TSM) from Himedia Laboratories, India. Incubation was carried out at 28 ± 2 °C for bacteria and 25 ± 2 °C for *Trichoderma* spp. Distinct bacterial colonies, exhibiting diverse morphological characteristics, were chosen, purified, and preserved in a 20% glycerol solution for future use. Fungal colonies were examined for morphological differences under a compound microscope at 400 × magnification (Olympus, Cx-21i, Japan). Subsequently, individual colonies identified as *Trichoderma* spp. were subcultured and stored based on their morphological features.

For isolating endophytic bacteria, healthy pigeonpea plants were harvested at the flowering stage. One gram stem samples underwent surface sterilization [70% alcohol (30 s), 1% sodium hypochlorite (30 s), sterile distilled water (3 × 60 s)], and were ground using a mortar and pestle in 9 mL of sterile water^32^. The grounded samples were serially diluted to 10^–8^, and 0.1 mL aliquots from this dilution were plated on Nutrient agar and King’s B agar plates. Incubation was done at 28 ± 2 °C in a BOD incubator for 2–3 days. (Supplementary Fig. 2).

### In vitro evaluation of fungal and bacterial biocontrol agents against *F*. *udum*

The dual culture technique was employed to evaluate the antagonistic effects of bacterial and fungal isolates against *F*. *udum* isolated from Pigeonpea cultivar ICP 8858. For fungal evaluation, 5 mm mycelial discs of seven days old *F*. *udum* were positioned on one side of a petriplate, while 5 mm discs of seven day old *Trichoderma* spp. fungal cultures were placed on the opposite end. These plates were then incubated for seven days at 25 ± 2 °C with three replications, and control plates were also included. As for the bacterial evaluation, 5 mm mycelial discs of the test pathogen were positioned at the center of PDA medium plates. Bacterial cultures were streaked on all four sides of the pathogen disc in a square pattern. Subsequently, these plates were incubated at 28 ± 2 °C for 7 days. Observations were made regarding the radial growth of the test pathogens with or without the presence of the antagonist, and the percentage of inhibition was calculated using the methodology outlined by^33^. The experiment was replicated for twice.

$${\text{I}}= \frac{{\text{C}}-\mathrm{T }}{\mathrm{C }}\times 100$$

I is the Per cent inhibition over control. C is the Radial growth of pathogen in control (mm). T is the Radial growth of pathogen in treatment (mm).

### Molecular identification of fungal and bacterial biocontrol agents

Based on their observed antagonistic activity, promising bacteria (Eb-8, Eb-11, Eb-13, Eb-21, Rb-4, Rb-11, Rb-14, Rb-18, and Rb-19) were selected and subjected to identification at the species level through 16S rRNA sequencing. Similarly, *Trichoderma* spp. were identified using TEF1α and ITS region gene sequencing. The CTAB method (Cetyl Trimethyl Ammonium Bromide), was utilized to extract total genomic DNA from both the bacteria and *Trichoderma* spp. Subsequently, the DNA pellet was dissolved in 50 μL of 1X TAE buffer, which consists of 10 mM Tris and 1 mM EDTA. DNA quantification was carried out on a 0.8% agarose gel, and purity was assessed by determining the A260/A280 ratio using a spectrophotometer. For amplifying the 16S rRNA gene of the bacterial isolates, forward primer (5′-GGATGAGCCHALGGCCTA-3′) and reverse primer (5′-CGGTGTGTACAAGGCCCGG-3′) were used. Subsequently, PCR reactions for *Trichoderma* spp. were performed using specific primer pairs, namely ITS for amplifying the Internal Transcribed Spacer region of Ribosomal DNA (ITS-rDNA) and Translation Elongation Factor 1-α gene (TEF1α). Eurofins Genomics in Bangalore, Karnataka, sequenced the amplified products using the Sanger sequencing method. Sequences were considered belonging to the same species when they were at least 99.7% identical, and those with at least 97.8% identity were classified as belonging to the same genus.

### Characterization and in vitro plant growth promoting activities of bacterial biocontrol agents

#### Biochemical characterization

A total of nine potential bacterial isolates, known for their antifungal properties against *F*. *udum*, underwent thorough biochemical characterization following the guidelines in Bergey's manual of determinative bacteriology. This involved a series of tests, including gram staining, amylase, catalase, oxidase, indole, methyl red, Voges–Proskauer, and citrate utilization tests^34^.

#### Plant growth promoting activities

##### Cellulase production test

The 24 h old bacterial isolates were inoculated on Carboxy Methyl Cellulose (CMC) agar medium plates and incubated at 28 °C for five days to allow the cellulase secretion. Following incubation, the agar medium was soaked in a congo red solution (1 per cent w/v) for 15 min. Subsequently, the congo red solution was drained and the plates were subjected to an additional treatment with 1 M NaCl for 15 min. The presence of a clearly identifiable hydrolysis zone indicated the degradation of cellulose^35^.

##### Siderophore production test

CAS (Chrome Azurol S) media was prepared and spot inoculation of the bacterial isolates was done from the actively growing cultures. Colonies that displayed an orange halo zone after 3 days of incubation at 28 ± 2 °C were regarded as positive for siderophore production^36^.

##### HCN and ammonia production tests

The method proposed^37^ was employed to assess the ability of bacteria to produce hydrogen cyanide. Each bacterium was streaked onto a nutrient agar medium containing 4.4 g/L of glycine. A Whatman no. 1 filter paper was placed over the agar, soaked in a specific solution (0.5% picric acid and 2% sodium carbonate w/v). The plates were sealed with parafilm and then incubated for 4 days at 36 ± 2 °C. The presence of an orange or red color indicated the formation of hydrogen cyanide.

The 24 h old bacterial cultures were inoculated in 10 mL of peptone broth and incubated at 28 ± 2 °C for 48–72 h. Later, one mL of Nessler’s reagent was added to each tube and the development of yellow to dark brown colour was taken as a positive reaction. Based on the intensity of colour, the isolates were divided into four groups i.e., + , +  + , +  +  + , +  +  +  + ^38^.

##### Phosphate, potassium, and zinc solubilization

The qualitative assessment of phosphate, potassium, and zinc solubilization activities of the isolates was conducted using specific agar media. For phosphate solubilization, pure colonies were spot inoculated onto Pikovskaya’s agar plates and then incubated at 28 ± 2 °C for 5 days. The confirmation of phosphate solubilization was based on the formation of a distinct halo zone around the colony^39^. Similarly, for potassium solubilization, isolates were spot inoculated onto Aleksandrov agar plates and incubated for 5 days. The presence of a clear halo zone around the colony indicated potassium solubilization^40^. In the case of zinc solubilization, isolates were spot inoculated onto Tris minimal agar medium supplemented with zinc oxide and then incubated at 30 °C for 3 days. The confirmation of zinc solubilization relied on the formation of a clear halo zone around the colony^41^. All experiments regarding biochemical tests Plant Growth Promoting Rhizobacteria (PGPR) activities were replicated for validation.

### Assessment of selected biocontrol agents against pigeonpea *Fusarium* wilt under pot conditions

Rhizosphere bacteria (Rb-18) and endophytic bacteria (Eb-21), exhibiting positive antifungal and Plant Growth Promoting Rhizobacteria (PGPR) activities, along with *Trichoderma* spp. isolated from the Pigeonpea rhizosphere, were selected as biocontrol agents. The experiment utilized seeds of the pigeonpea wilt susceptible cultivar (ICP 2376).

The experimental setup involved pot cultivation using sterilized pots measuring (20 × 15) cm. Each pot was filled with 5 kg of sterilized sandy loamy soil, and 10 surface sterilized seeds were sown for each treatment, with three replications. After 35 days of sowing, five pots were inoculated with a spore suspension of *F*. *udum* (50 mL of microconidial suspension containing 1 × 10^6^ conidia/mL per pot). Among these, three pots were inoculated with a *Trichoderma* spp. spore suspension (6 mL) (1 × 10^6^ spores/mL), and two pots with a bacterial suspension (10 mL of a suspension containing 10^8^ cfu/mL) on the 45th day. Plants that were inoculated with the pathogen and those not treated with either the pathogen or biocontrol agents served as control groups. The greenhouse experiment was conducted under high humidity (≥ 90%) and optimal temperature conditions of 28–30 °C. Each treatment was replicated three times in a completely randomized design.

The per cent disease incidences was calculated by the following formula

$$ {\text{PDI }} = \frac{{{\text{No}}.{\text{ of wilted plants}}}}{{{\text{Total no}}.\,{\text{of plants}}}} \times 100 $$

### Activity of defence enzymes in biocontrol treated plants against Pigeonpea *Fusarium* wilt

The study evaluated the activity of defense related enzymes, including peroxidase (POD), polyphenol oxidase (PPO), and phenylalanine ammonia lyase (PAL), in Pigeonpea plants treated with *Trichoderma* spp. and bacterial biocontrol agents when challenged with *F*. *udum* under potted conditions. Fresh leaves were collected randomly from each treatment at different time points: 0, 24, 48, 72 and 96 h after the inoculation with biocontrol agents. The leaf tissues were immersed in liquid nitrogen and homogenized in 10 mL of ice cold 50 mM potassium phosphate buffer (pH 6.8) containing 1 M NaCl, 1 mM EDTA, 1% polyvinyl pyrolidone and 10 mM β-mercaptoethanol. The samples were filtered using muslin cloth and centrifuged at 12,000 rpm at 4 °C for 25 min. The final supernatants were used for the assay of peroxidase and polyphenol oxidase enzymes. The standard assay protocol described by^21^ was followed for peroxidase and polyphenol oxidase. To determine PAL activity, 400 µL of sample extract was incubated with 0.5 mL of 0.1 M borate buffer pH 8.8 and 0.5 mL of 12 mM l-phenylalanine in the same buffer for 30 min at 30 °C. PAL activity was determined as the rate of conversion of l-phenylalanine to transcinnamic acid at 290 nm. The amount of trans-cinnamic acid synthesised was calculated using its extinction coefficient of 9630 M^−1^ cm^−1^. Enzyme activity was expressed in fresh weight basis as nmol trans-cinnamic acid min^−1^ mg^−1^ of sample^42^.

### Assessment of selected biocontrol agents against Pigeonpea *Fusarium* wilt under sick plot conditions

The study was conducted at the AICRP on Pigeonpea wilt sick plot located at T.C. A Dholi, R.P.C.A.U (25° 59′ 41.9″ N 85° 35′ 43.3″ E), Pusa, Bihar. The experiment was carried out over four different seasons, which included Kharif 2021–2022, Rabi 2021–2022, Kharif 2022–2023, and Rabi 2022–2023. To ensure even distribution of the pathogen within the affected plots, four soil samples were taken from each season (3 m × 3 m) plot. These samples underwent a series of dilutions and were then plated on a specialized *Fusarium* medium following the method outlined by^43^.

The *B*. *subtilis* isolates were inoculated into nutrient broth, while *P*. *aeruginosa* isolates were introduced into KB broth. The cultures were then incubated at 28 ± 2 °C 28 ± 2 °C for 36 h on a rotary shaker set at 150 rpm. After incubation, the bacteria were collected through centrifugation at 8000 rpm for 10 m using a benchtop refrigerated centrifuge. The resulting pellets were washed three times with sterile distilled water (SDW) and the cell concentration was adjusted to 1 × 10^8^ colony forming units (cfu) per millilitre through dilution, aiming for suspensions with an optical density of 0.45 at A610 nm, as determined by a UV–visible spectrophotometer (Mortensen, 1992). The *Trichoderma* spp. isolates were cultured on PDA plates for 10–12 days at 28 ± 2 °C. Subsequently, 10 mL of sterile distilled water (SDW) was added to each plate, and conidia were gently detached from the culture surface by shaking. The remaining conidia were removed using a sterile brush, and the resulting suspension was collected in a 100 mL conical flask. After passing the conidial suspension through four layers of cheesecloth, it was centrifuged at 2500 rpm for 10 min and then resuspended in distilled water. The conidial concentration was adjusted to 1 × 10^6^ conidia per millilitre using a hemocytometer.

Pigeonpea seeds of wilt susceptible cultivar ICP8863 were soaked in a culture suspension with the addition of 0.2% carboxymethyl cellulose (CMC) to aid in the attachment of the biocontrol agent to the seeds. These treated seeds were then incubated at 28 ± 2 °C in a rotary shaker at 150 rpm for 6 h and subsequently air dried under sterile conditions. While carnbendizim was treated as 2.0 mg/g seeds. As a control, seeds soaked in distilled water amended with 0.2% CMC were used. These treated seeds were manually sown in wilt affected plots with a spacing of 90 cm between rows and 20 cm within rows, at a depth of 2–3 cm. The experimental design followed a randomized block pattern with seven treatments, each replicated. Each replication occupied a 3 m × 3 m plot, totalling an area of 9 square meters. The incidence of wilt was assessed 65 days after sowing.

The per cent disease incidences was calculated by the following formula

$$ {\text{PDI }} = \frac{{{\text{No}}.{\text{ of wilted plants}}}}{{{\text{Total no}}.{\text{ of plants}}}} \times 100 $$

### AMMI analysis

In this study, the performance of seven Treatments (T) and their interactions with four Environments (E) were assessed. Disease incidence data collected from the treatments were organized to be compatible with the AMMI (Additive Main Effects and Multiplicative Interaction) models. The AMMI statistical model, along with computational methods detailed in^44^, was employed for the analysis. An analysis of variance was conducted to partition the variation into main effects associated with the Treatments (T) and the Environments (E), as well as the interaction effect between Treatments and Environments (T × E). These analyses were carried out using the GEA-R software developed by 'CIMMYT' and the 'R' package Agricolae.

### Ethical statement

All authors have approved the manuscript and agreed with its submission to the Scientific Reports. The submitted work is original and has not been submitted or published elsewhere. The manuscript has been prepared following principles of ethical and professional conduct. The study does not involve human participants or animals.

### IUCN policy statement

The experimental research and field studies on plants, both cultivated and wild, strictly followed institutional, national, and international guidelines, including the IUCN Policy Statement on Research Involving Species at Risk of Extinction and the Convention on the Trade in Endangered Species of Wild Fauna and Flora. Emphasizing our commitment to ethical research, no endangered species of wild fauna and flora were involved, reflecting our dedication to biodiversity conservation and minimizing adverse impacts on vulnerable plant populations. This comprehensive compliance aims to advance scientific knowledge while championing environmental sustainability and global biodiversity preservation, upholding the highest standards of research integrity for the well-being of ecosystems and future generations.

## Results

### Morphological, pathogenic and molecular characterisation of the pathogen

**Figure 1:**
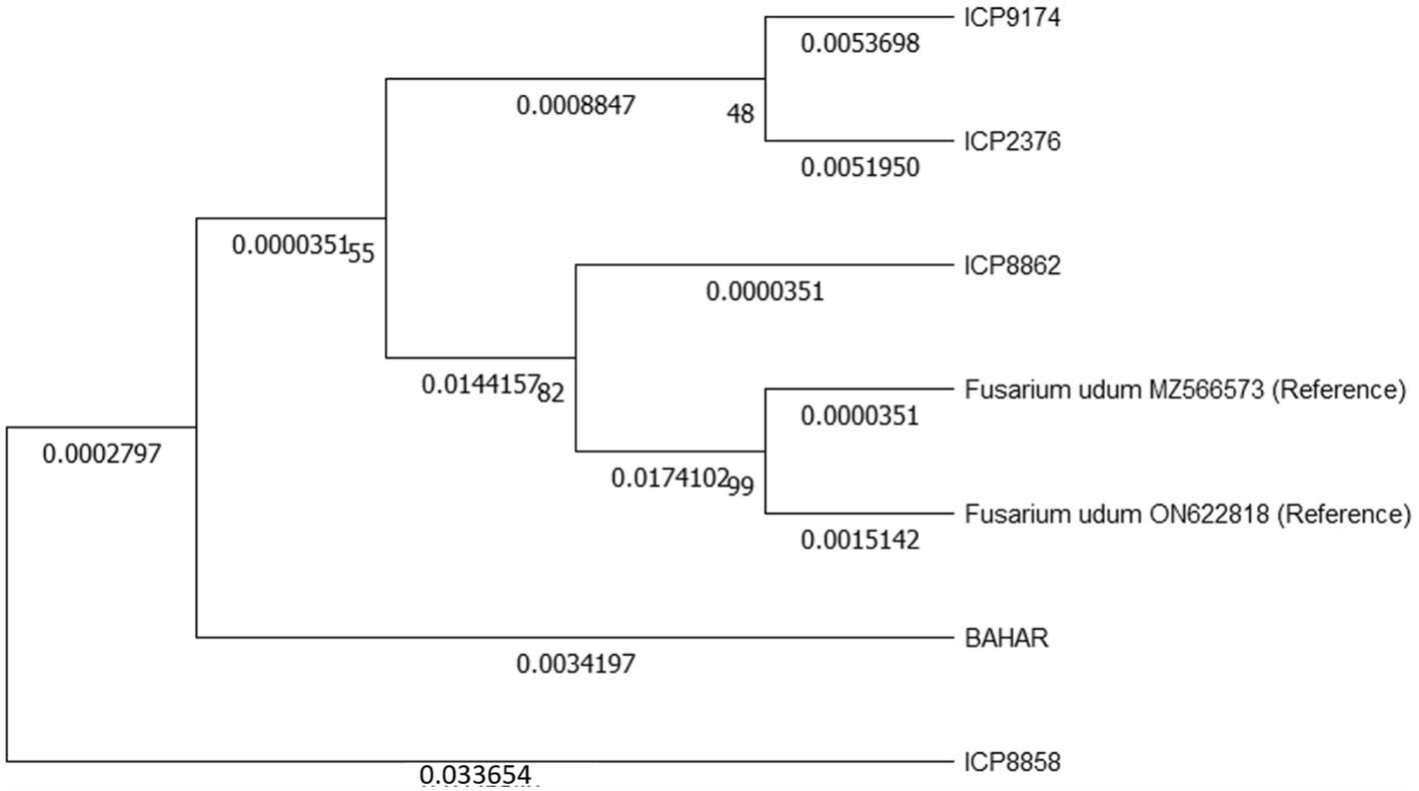
Multiple sequence alignment of ITS and TEF genes of *Fusarium* isolates using Maximum Likelihood method with 1000 boot strap values.

In the present study, a total of five *Fusarium* isolates were obtained, each originating from a distinct Pigeonpea cultivar (ICP 2376, BAHAR, ICP 8862, ICP 8858, and ICP 9174). The cultural and morphological traits of these *Fusarium* isolates were investigated on PDA, revealing notable differences in colony texture, substrate pigmentation, mycelial color, and conidia length and width (Supplementary Fig. 3). All *Fusarium* isolates exhibited pathogenicity in causing wilt disease during the pathogenicity test, with an incidence ranging from 60 to 90%. Notably, the *Fusarium* isolate obtained from the ICP 8858 cultivar demonstrated the highest disease incidence at 90%, indicating its virulence and was subsequently chosen for further antagonistic investigations. To molecularly characterize these isolates, PCR amplification of the ITS-rDNA region using universal primers yielded amplicons ranging from 500 to 550 bp in length. Additionally, an analysis of nucleotide sequences of the TEF1α gene revealed variations in length, ranging from 670 to 725 base pairs among the five *Fusarium* isolates. Subsequently, all sequences were submitted to the NCBI GenBank, and accession numbers were obtained for reference and documentation purposes (Table 1) (Fig. 1).

**Table 1:** Cultural and morphological characters of *F. udum* isolates.

S.no	Cultivar	Radial growth rate (mm)	Mycelium growth pattern	Mycelia colour	Substrate pigmentation	Zonation’s	Macro conidia (µm)	Micro conidia (µm)	Disease incidence (%)	ITS gene accession numbers	TEF gene accession numbers
1	ICP 2376	86	Moderately Fluffy	White	Buff	Absent	24.26 × 2.12	8.22 × 3.22	60	OR267399	PP060448
2	BAHAR	80	Fluffy	Buff	Buff	Absent	27.45 × 3.99	11.68 × 2.9	75	OR267401	PP060447
3	ICP 8862	80	Appressed	Off White	Yellowish white	Absent	32.17 × 2.32	9.80 × 2.67	80	OR267402	PP060449
4	ICP 8858	86	Fluffy	White	White	Absent	31.17 × 3.53	10.88 × 3.44	90	OR083610	PP060445
5	ICP9174	80	Appressed	Mauve	Plum	Present	22.17 × 3.52	8.88 × 2.65	70	OR267395	PP060446

### Isolation of beneficial microbes

In our present study, based on cultural and morphological traits a total of 100 endophytic and 100 rhizosphere bacteria were isolated, purified and evaluated for antagonistic activity against *F. udum*. Simultaneously, we isolated three *Trichoderma* strains from 10 rhizosphere soil samples and compared them to the *Trichoderma* Taxonomy database https://www.ncbi.nlm.nih.gov/Taxonomy/Browser/wwwtax.cgi?id=5543 using criteria like conidiospore color and pigment secretion on the PDA medium. Subsequent microscopic examination confirmed the presence of three isolates: *T*. *harzianium*, *T*. *asperellum*, and an unidentified *Trichoderma* species. Importantly, two of these isolates, *T*. *harzianium* and *T*. *asperellum*, were categorized within the *Harzianum* clade and *Hamatum* sub branch, respectively, while the third isolate, *Trichoderma* sp., could not be conclusively identified.

### In vitro evaluation of biocontrol agents against *F*. *udum*

**Figure 2:**
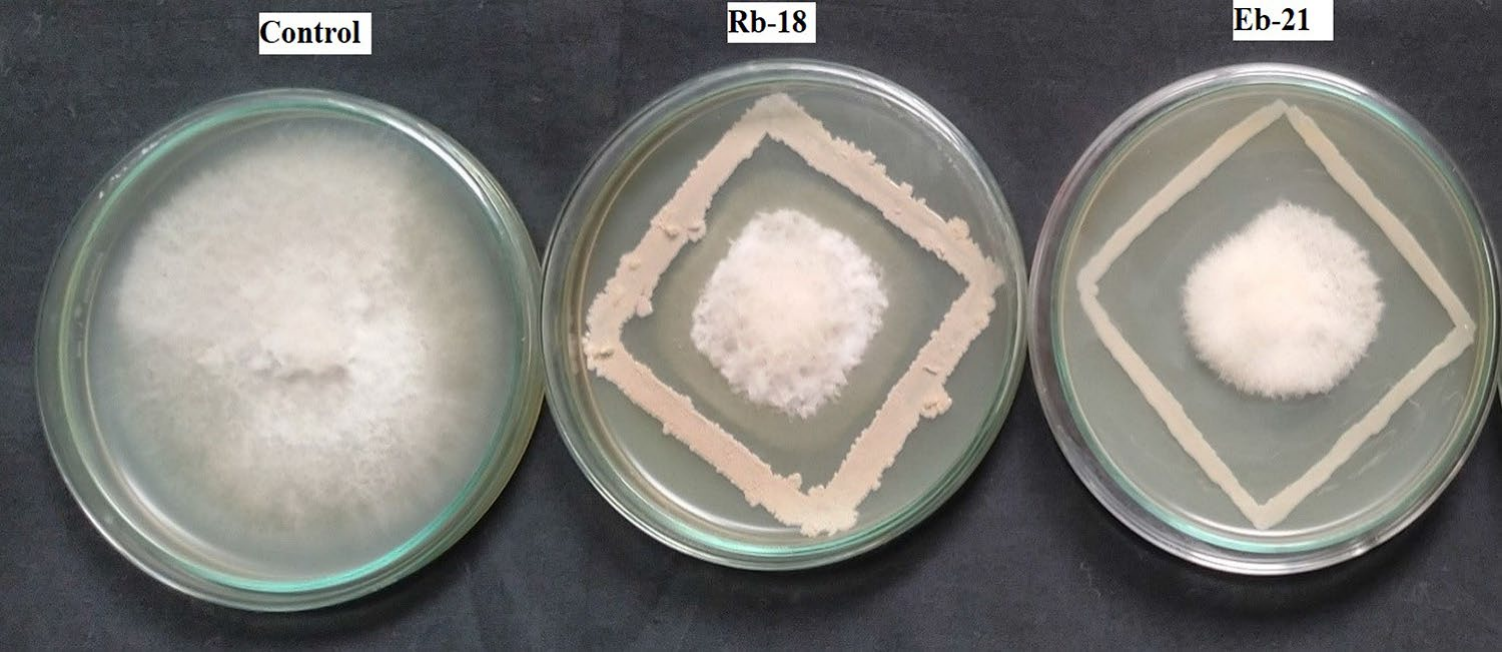
Antagonistic activity of bacterial isolates against *F. udum*: RB-18 (Rhizosphere bacteria) and Eb-21 (Endophytic bacteria**).**

In the dual culture technique, it was noted that among the tested bacterial isolates, four endophytic and five rhizosphere isolates effectively inhibited the growth of *F*. *udum* by more than 60%. Specifically, the endophytic bacterial strains identified as Eb-21, Eb-13, Eb-8, and Eb-11 exhibited inhibition percentages of 72.22%, 65.11%, 64.44%, and 62.88%, respectively. In contrast, rhizosphere bacteria labeled as Rb-18, Rb-14, Rb-19, Rb-4, and Rb-11 exhibited inhibition percentages of 71.11%, 68.44%, 65.3%, 64.8%, and 62.11%, respectively (Fig. 2). *T*. *harzianum*, *T*. *asperellum*, and *Trichoderma* sp. exhibited inhibition percentages of 65%, 60%, and 55%, respectively, against *F*. *udum*.

### Molecular based identification of bacterial and fungal isolates

**Figure 3:**
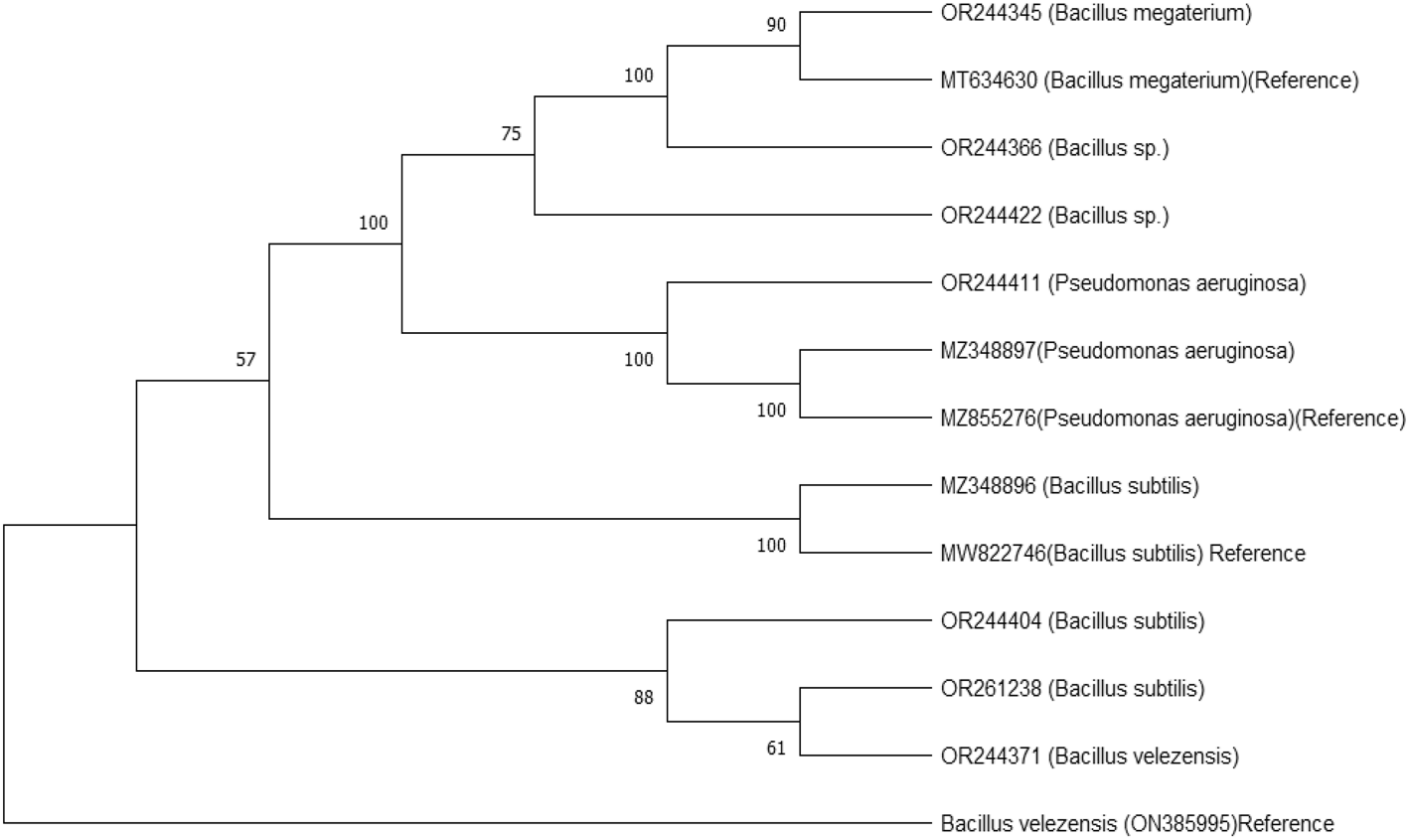
Phylogenetic tree for 16S rRNA gene of bacterial isolates using neighbour-joining method.

Based on their antifungal characteristics, nine bacterial strains and three *Trichoderma* species were selected for molecular identification. The Polymerase Chain Reaction (PCR) method was utilized to amplify fragments of the bacterial 16S rRNA gene. Subsequently, the obtained 16S rRNA gene sequences were compared against the NCBI nucleotide database using the Basic Local Alignment Search Tool (BLAST). The results of this comparison led to the identification of the isolates as follows: Rb-4 (*Bacillus* sp.), Rb-11 (*B*. *subtilis*), Rb-14 (*B*. *megaterium*), Rb-18 (*B*. *subtilis*), Rb-19 (*B*. *velezensis*), Eb-8 (*Bacillus* sp.), Eb-11 (*B*. *subtilis*), Eb-13 (*P*. *aeruginosa*), and Eb-21 (*P*. *aeruginosa*). The genetic sequences were subsequently deposited into the NCBI GenBank, and specific accession numbers were obtained (Fig. 3, Table 2).

**Table 2:** Biochemical and molecular characterization of bacterial isolates.

SI. No	Isolates	Grams reaction	Amylase test	Catalase test	Oxidase test	Indole test	Methyl red test	Voges-Proskauer test	Citrate test	Accession numbers	Organism identified
1	Eb-8	+	−	+	−	−	+	−	−	OR244422	*Bacillus* sp*.*
2	Eb-11	** + **	−	+	+	−	−	−	−	OR261238	*B. subtilis*
3	Eb-13	−	+	+	−	−	+	−	+	OR244411	*P. aeruginosa*
4	Eb-21	−	+	+	+	−	−	−	−	MZ348897	*P. aeruginosa*
5	Rb-4	+	+	+	+	−	−	−	+	OR244366	*Bacillus* sp.
6	Rb-11	+	−	+	+	−	−	−	−	OR244404	*B. subtilis*
7	Rb-14	+	+	+	+	−	−	−	−	OR244345	*B. megaterium*
8	Rb-18	+	+	+	+	−	−	−	−	MZ348896	*B. subtilis*
9	Rb-19	+	+	+	−	−	−	−	−	OR244371	*B. velezensis*

**Figure 4:**
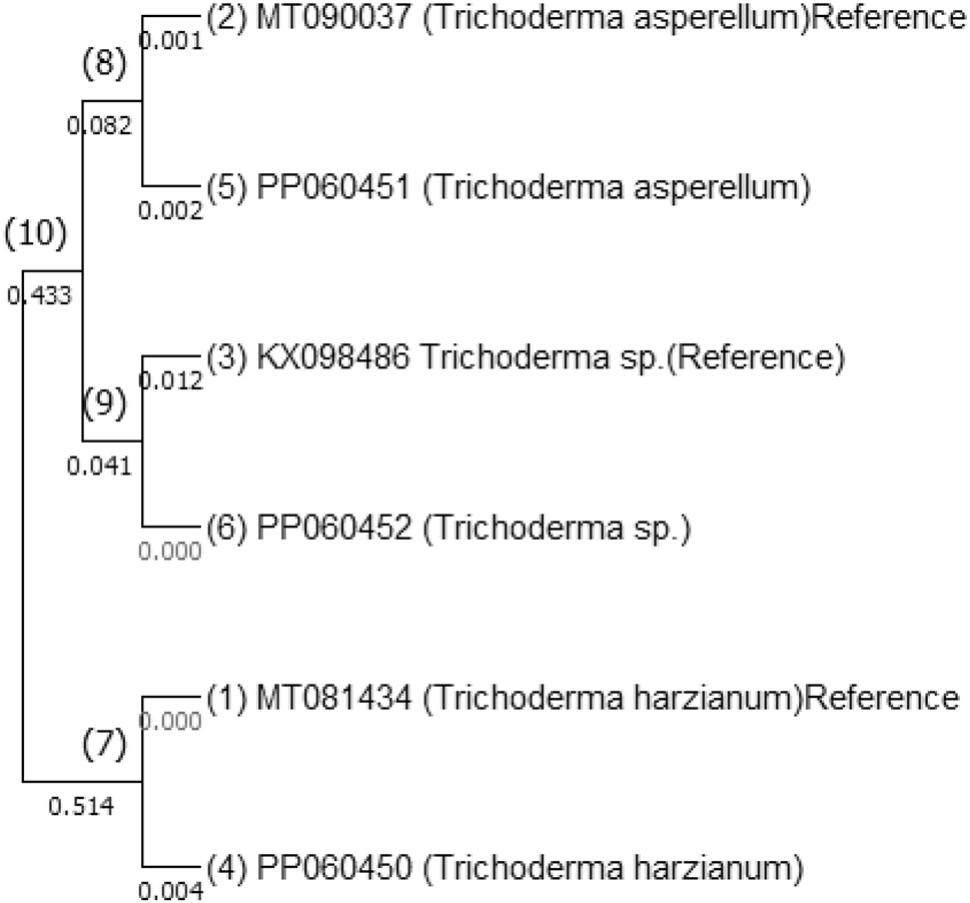
Multiple sequence alignment of ITS and TEF genes of *Trichoderma* isolates using Maximum Likelihood method with 1000 boot strap values.

Similarly, for the *Trichoderma* isolates, BLAST analysis was employed to compare their fungal TEF (Translation Elongation Factor 1-α gene) and small ribosomal gene (18S rRNA gene) sequences with existing *Trichoderma* sequences in the NCBI database. The BLAST analysis confirmed that the amplified TEF and ITS gene sequences from the *Trichoderma* isolates showed similarity to known *Trichoderma* species. Consequently, the sequences were submitted to the NCBI GenBank, securing accession numbers: ITS (MZ348898) TEF (PP060450) for *T*. *harzianum*, ITS (MZ411690) TEF (PP060451) for *T*. *asperellum*, and ITS (MZ411691) TEF (PP060452) for *Trichoderma* sp. (Fig. 4).

### Biochemical characterization of bacterial isolates

Bacterial isolates that demonstrated inhibitory effects on *F*. *udum* in dual culture experiments underwent biochemical characterization. Among these isolates, all tested positive for the catalase test, seven displayed a positive gram reaction, six exhibited positive results for amylase and oxidase tests and two indicated positive outcomes for citrate utilization and methyl red reduction tests. However, none of the isolates showed a positive result in the indole production test (Table 2).

### In vitro plant growth promoting activities

A total of nine potential bacterial isolates, which exhibited inhibitory effects against *F*. *udum* in a dual culture technique, underwent in vitro assessment for their growth promoting activities. The cellulase activity of these potential bacterial isolates was evaluated using CMC agar media. The presence of a halo zone around the colony was considered a positive outcome for this test, and variations were observed among the isolates. Specifically, four isolates, namely Eb-8, Eb-21, Rb-14, and Rb-18, exhibited halo zones around their colonies. None of the isolates showed hydrogen cyanide (HCN) production. Interestingly, it was noted that the rhizosphere bacterial population (Rb-18) displayed a higher capacity for siderophore production compared to the endophytic bacteria (Eb-21). Ammonia production was recorded in three isolates Eb-21, Rb-11 and Rb-18.

Additionally, bacterial isolates demonstrating the ability to solubilize inorganic phosphate, potassium, and zinc were assessed based on the formation of clear halo zones in Pikovaskaya’s, Aleksandrov, and Trisminimal agar plates, respectively. In Pikovaskaya’s medium, isolates Eb-21, Rb-14, and Rb-18 exhibited the formation of halo zones (Supplementary Fig. 4). Similarly, on Aleksandrov agar plates, Rb-11 and Rb-18 displayed a halo zone, and on zinc supplemented Trisminimal agar, Eb-21, Rb-11, Rb-14, and Rb-18 exhibited halo zones (Table 3).

**Table 3:** In vitro screening of biochemical and enzymes of biocontrol importance: The efficient isolates with biocontrol potential were screened for cellulose, HCN, Siderophores, ammonia production and Phosphorus, Potassium and Zinc solubilisation.

S. No	Isolate	Cellulase	HCN	Siderophore	Ammonia	Phosphorus	Potassium	Zinc
1	Eb-8	** + **	−	−	−	−	−	−
2	Eb-11	−	−	−	−	−	−	−
3	Eb-13	−	−	−	−	−	−	−
4	Eb-21	** + + **	−	** + **	** + **	** + **	−	** + + **
5	Rb-4	−	−	** + + **	−	−	−	−
6	Rb-11	−	−	** + **	** + **	−	** + **	** + **
7	Rb-14	** + + **	−	** + **	−	** + **	−	** + **
8	Rb-18	** + + **	−	** + + + **	** + **	** + + **	** + **	** + + **
9	Rb-19	−	−	−	−	−	−	−

### Assessment of selected biocontrol agents against Pigeonpea *Fusarium* wilt under pot conditions

The potted plants experiment aimed to evaluate the effectiveness of various biocontrol agents, namely *B*. *subtilis*, *P*. *aeruginosa*, *T*. *harzianum*, *T*. *asperellum*, and *Trichoderma* sp., in reducing *Fusarium* wilt in Pigeonpea. The disease incidence in the control group without any treatment (T6) was high at 93.33%. However, the treatment involving *P*. *aeruginosa* and *F*. *udum* (T2) exhibited the lowest disease incidence at 20%. This was followed by the treatments with *T*. *harzianum* + *F*. *udum* (T3) at 21.66%, *B*. *subtilis* + *F*. *udum* (T1) at 23.33%, *T*. *asperellum* + *F*. *udum* (T4) at 26.66%, and *Trichoderma* sp. + *F*. *udum* (T5) at 29.33% (Table 4).

**Table 4:** Evaluation of promising biocontrol agents against Pigeonpea *Fusarium* wilt in pot conditions.

S.no	Treat no	Treatments	Mean disease incidence
1	T1	*B*. *subtilis* + *F*. *udum*	23.33^d^
2	T2	*P*. *aeruginosa* + *F*. *udum*	20^b^
3	T3	*T*. *harzianum* + *F*. *udum*	21.66^c^
4	T4	*T*. *asperellum* + *F*. *udum*	26.66^e^
5	T5	*Trichoderma* sp. + *F*. *udum*	29.33^f^
6	T6	*F*. *udum*	93.33^g^
7	T7	Control	0^a^
CD (p = 0.05) 1.512			

Means with same letters between treatments are not significantly different (LSD at p = 0.05).

### Activity of defence enzymes in biocontrol treated plants against Pigeonpea *Fusarium* wilt

In this study, the enzymes associated with plant induced systemic resistance (ISR), including peroxidase (POD), polyphenol oxidase (PPO) and phenylalanine ammonia lyase (PAL), were investigated in vitro. Prospective biocontrol bacteria and *Trichoderma* spp. isolates were introduced to the plants. The results of the study showed that the highest levels of peroxidase (POD) and polyphenol oxidase (PPO) activity were observed in plants treated with *P*. *aeruginosa* + *F*. *udum* (1.53) (POD), 1.53 (PPO) and (27) (PAL)) followed by *B*. *subtilis* + *F*. *udum* and *T. harzanium* + *F. udum*. Notably, the POD, PPO, and PAL activity levels were significantly higher in plants treated with bacteria compared to those treated with fungi. Enzyme activity showed a notable increase in all treatments, peaking at 72 h before gradually declining. Control plants, which were neither exposed to the pathogen nor the biocontrol agents, exhibited consistent enzyme activity levels across all time intervals. In contrast, plants treated with the pathogen did not display any significant POD, PPO, or PAL activity when compared to plants treated with the biocontrol agents (Supplementary Figs. 5, 6, 7).

### Assessment of selected biocontrol agents against pigeonpea *Fusarium* wilt under sick plot conditions

**Figure 5:**
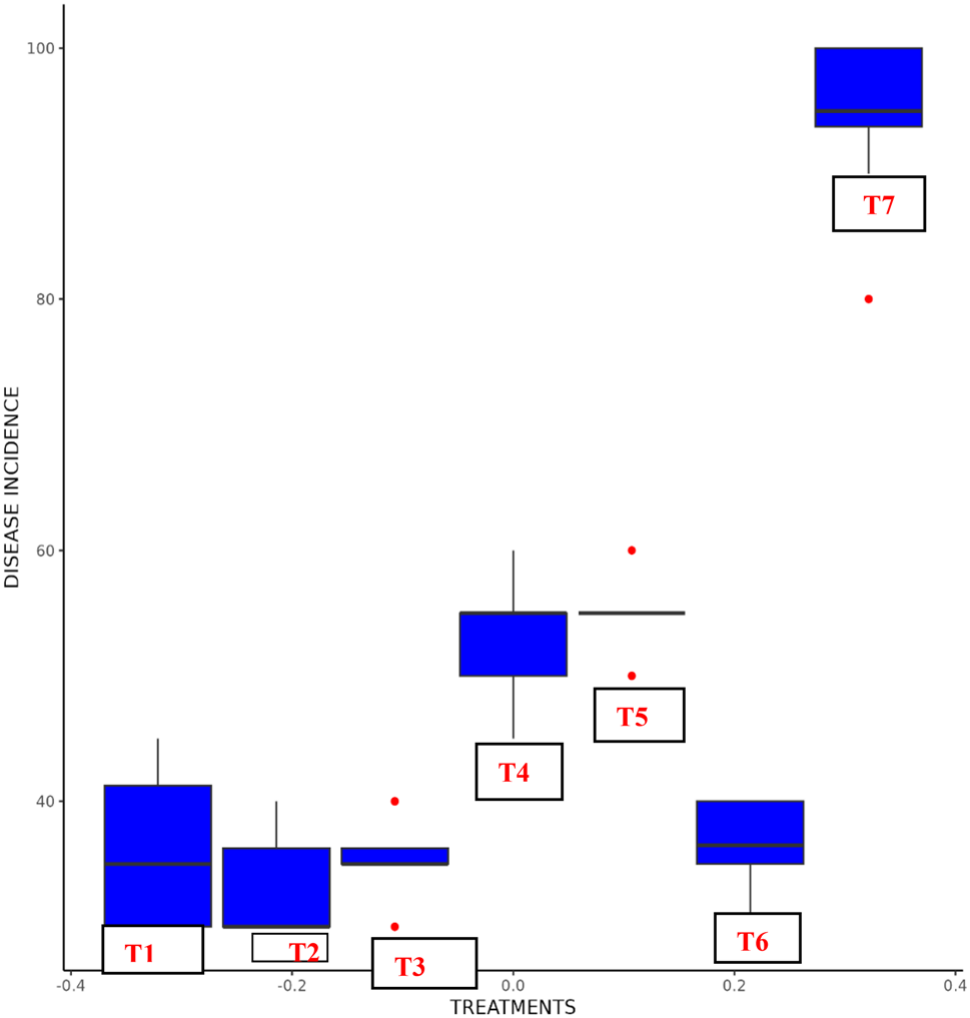
Disease incidence of promising biocontrol agents in Pigeonpea *Fusarium* wilt sick plot.

The potential fungal and biocontrol agents were applied as seed treatments on the wilt susceptible cultivar ICP2376 and evaluated for their effectiveness against pigeonpea wilt in sick plots over four seasons (2021–2022 Kharif, 2021–2022 Rabi, 2022–2023 Kharif, 2022–2023 Rabi). In all treatments during these four seasons, the lowest mean incidence of the disease was observed in T2 (33.33) (*P*. *aeruginosa*) and T3 (35.41) (*T*. *harzanium*) followed by T6 (36.5) (Carbendizim), T1 (36.66) (*B*. *subtilis)*, T4 (52.91) (*T*. *asperellum*) and T5 (53.33) (*Trichoderma* sp.) (Table 5; Fig. 5).

**Table 5:** Disease incidence of promising biocontrol agents against Pigeonpea *Fusarium* wilt in sick plot.

S.no	Treatments	Kharif 2021–2022	Rabi 2021–2022	Kharif 2022–2023	Rabi 2022–2023	Mean disease incidence
1	*B. subtilis* + *F. udum* (T1)	41.67	31.67	38.33	35	36.66^a^
2	*P. aeruginosa* + *F. udum* (T2)	33.33	30	35	35	33.33^a^
3	*T. harzanium* + *F. udum* (T3)	36.67	35	33.33	36.67	35.41^a^
4	*T. asperellum* + *F. udum* (T4)	53.33	53.33	51.67	53.33	52.91^b^
5	*Trichoderma* sp. + *F. udum* (T5)	56.67	53.33	53.33	55	54.58^b^
6	Carbendizim seed treatment (T6)	36.67	33.33	38.33	37.67	36.5^a^
7	*F. udum* (T7)	91.67	95	98.33	91.67	94.16^c^
CD (p = 0.05) 3.728						

Means with same letters between treatments are not significantly different (LSD at p = 0.05).

#### AMMI ANNOVA

ANOVA of seven Treatments (T) over four Environments (E) showed that 0.24% of the total SS was attributed to Environments (E) effect; 95.08.% to Treatments (T) effects and 0.88% to Treatments by Environments (T × E) interaction effects. The T × E was further divided into Interaction Principal Component Axis (IPCA) and residuals, in which IPCA1 has contributed (49.01%) of interaction SS followed by IPCA2 which contributed (37.03%) of interaction SS and IPCA1 and IPCA2 cumulatively contributed to (97.411%) of the total interaction (Table 6).

**Table 6:** AMMI ANNOVA for biocontrol agent’s treatments × environments interactions.

S.no	Source	Df	Sum Sq	% Sum of suares	Mean Sq	F value	P
1	Environment	3	88.41667	0.24	29.47222	0.455169	S
2	Treatment	6	33,897.67	95	5649.611	327.5137	S
4	Treatment: environment	18	316.3333	0.88	17.57407	1.018787	S
5	PC1	8	154.9392	49.01	19.3674	1.12	S
6	PC2	6	117.1309	37.03	19.52181	1.13	S
7	Residuals	48	828	2.32	17.25	NA	
8	Total	101	35,964.75		356.0866	NA	

*Df* Degree of freedom, *SS* Sum of squares, *MSS* Mean sum of squares, *IPCA* Interaction Principal Components Axis, *F* F calculated value, *P* Probability, *S* Significant.

#### AMMI 1 Biplot display

**Figure 6:**
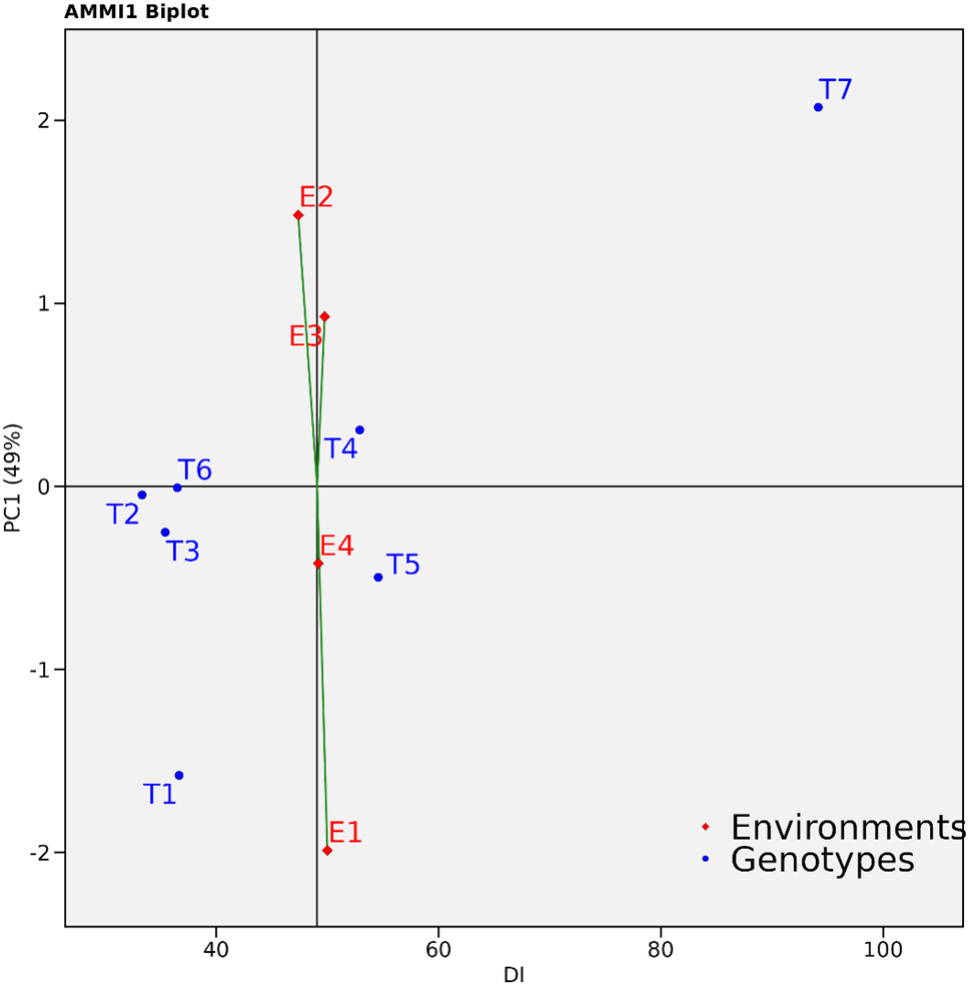
AMMI1 biplot displaying disease incidence and IPCA1 scores of promising biocontrol agents over four environments.

The AMMI1 biplot was employed to analyze the average disease incidence and IPCA1 scores of seven treatments in four different environments. It revealed that treatments on the left side of the perpendicular line exhibited lower disease incidence, with T2 having the lowest, followed by T3 and T1. Conversely, treatments on the right side of the perpendicular line displayed higher disease incidence, with T6 having a particularly higher incidence (Fig. 6).

## Discussion

*Fusarium* wilt, caused by the fungus *F*. udum, poses a significant threat to pigeonpea cultivation worldwide, leading to substantial yield losses^3,4^. *F*. *udum* persists in the soil for extended periods through the formation of chlamydospores and acts as a hemibiotroph when it resides on infected plant remains ^2,5^. The prolonged persistence of the fungus in the soil and plant debris hampers disease management using conventional methods such as crop rotation and flooding ^45,46^. Currently, chemical control methods are commonly employed to address this serious wilt disease^11^. While fungicide application has proven helpful up to seed treatment, it is neither feasible nor economical for crops in the field due to the soil borne nature of *F*. *udum*^45^. Moreover, there is a possibility of the pathogen developing resistance to commonly used fungicides^15^. Environmental safety concerns also drive the exploration of alternative management strategies that are sustainable in the long run. Although certain resistant pigeonpea cultivars against *Fusarium* wilt have been identified previously, questions remain regarding the durability of field resistance to *F*. *udum* infection over time under field conditions^47^. Additionally, challenges arise from the evolution of new pathogen variants, the presence of location specific isolates, and the physiological specialization within the *Fusarium* sp. complex, which hinder successful wilt disease management in pigeonpea. Earlier studies on pathogenic variability in pigeonpea wilt have reported three different pathogenic groups^48^, five pathogenic variants^30^, and nine variants^7^. While soil solarization can address some of these challenges, it has adverse effects on soil quality and beneficial microorganisms^49^. Biological control emerges as an alternative approach to combat soil borne diseases^50^.

Biocontrol agents sourced from the native rhizosphere and within plant tissues are preferred due to their adaptability to local soil and climatic conditions^51^. Moreover, the composition of beneficial microbial populations in the rhizosphere is influenced by both plant root exudates and soil characteristics^52^. However, the fertile alluvial soils rich in organic matter found in Samastipur and Muzaffarpur districts of Bihar, influenced primarily by sediment deposition from the Gangetic alluvium in the Indo-Gangetic plains, support the growth of bioagents capable of effectively managing wilt diseases and promoting plant growth. Consequently, our recent study aimed to investigate the potential of native microflora isolated from various rhizosphere zones in Bihar for the biocontrol of *Fusarium* wilt in pigeonpea, as well as for enhancing plant growth. In our study, we assessed 100 endophytic bacteria, 100 native rhizosphere bacteria, and three *Trichoderma* spp. against *F*. *udum*. Among these, four endophytes, five rhizosphere bacteria, and three *Trichoderma* spp. exhibited inhibition rates exceeding 60% compared to the control, indicating their potential as promising isolates. Similar findings were reported by^32,53^, who observed that endophytic and rhizosphere bacteria effectively suppressed *F*. *udum* growth by inhibiting mycelial development and spore germination. Consistent with our results^11^ reported that rhizobacteria from pigeonpea demonstrated fungicidal effects against *F*. *udum*. This fungicidal activity was attributed to the synthesis of various biocidal substances, including antifungal metabolites, chitinolytic compounds, enzymes capable of breaking down cell walls, and volatile compounds with antifungal properties like ammonia and cyanide.

In laboratory conditions, it was observed that certain rhizobacteria, namely Rb-4, Rb-11, Rb-14, Rb-18, and the endophytic bacterium Eb-21, demonstrated the capability to produce siderophores. In natural soil environments, the production of siderophores is more prevalent among the rhizobacterial community^54^. The synthesis of siderophores by rhizobacteria plays a crucial role in their capacity to regulate the growth of pathogens. This is achieved by diminishing the availability of ferric ions in the rhizosphere, effectively inhibiting the growth and virulence of soil borne plant pathogens. An illustrative example of this phenomenon is seen in *P*. *aeruginosa*, which, when capable of producing siderophores under laboratory conditions, exhibits a broad spectrum of antagonistic effects against pathogens like *F*. *ciceri* and *F*. *udum*^55,56^. Similarly, research has indicated that strains of *B*. *atrophaeus* and *B*. *subtilis*, proficient in siderophore production, can effectively suppress the growth of wilt disease causing pathogens in crops such as cotton (*Fusarium* oxysporum)^57^ and pepper^58^ both under in vitro and in vivo conditions.

Plant Growth Promoting Rhizobacteria (PGPR) possess the ability to produce compounds like hydrogen cyanide (HCN) and ammonia (NH3), which play a dual role in inhibiting fungal growth and promoting plant development^59,60^. Notably, the ammonia produced by PGPR disperses in the soil, effectively eliminating infectious propagules of specific plant pathogens^61^. Additionally, it serves as a nitrogen source for host plants, facilitating the growth of roots and shoots, ultimately increasing overall biomass^62,63^. In our current study, three bacterial isolates, namely Eb-21, Rb-11, and Rb-18, exhibited positive ammonia production. These results align with previous findings on NH3 production by rhizospheric strains of *Bacillus* and *Pseudomonas* under in vitro conditions. Furthermore, these strains effectively managed disease incidence caused by *F*. *udum* in in vivo conditions^11^. However, it is important to note that all nine isolates tested negative for the HCN test in this study. In a related investigation by^64^, it was documented that two rhizosphere strains of *B*. *subtilis* and two endophytic bacterium strains of *P*. *aeruginosa* also exhibited an inability to produce HCN. Furthermore, biocontrol agents employ critical mechanisms such as cell wall-degrading enzymes, notably cellulase, to regulate soilborne pathogens^65^. Cellulase exhibits a potent inhibitory effect on the hyphal growth of fungal pathogens by hydrolyzing the 1,4-β-d-glucosidic linkages in cellulose, playing a significant ecological role in recycling cellulose, a major polysaccharide in nature^66,67^. This degradation process involves various cellulolytic enzymes such as cellulases/endoglucanases, exo-glucanases, and β-glucosidases, which synergistically convert cellulose into β-glucose. In our study, bacterial isolates Eb-8, Eb-21, Rb-14, and Rb-18 exhibited positive cellulase production, consistent with previous findings indicating that biocontrol agents produce lytic enzymes and cellulase to degrade pathogen cell walls^68^. Similarly, research by^11,69^ has demonstrated the inhibitory effects of cellulases produced by bacteria from the *Bacillus* and *Pseudomonas* genera on the growth of phytopathogenic fungi, thereby contributing to disease suppression in chickpea and pigeonpea wilt.

Phosphorus (P), Potassium (K), and zinc (Zn) are essential macronutrients crucial for biological growth and development. However, the concentrations of soluble P, K, and Zn in the soil are typically low because the majority of these nutrients exist in insoluble forms within rocks, minerals, and other deposits^70,71^. PGPR play a crucial role in mobilizing these nutrients in the rhizosphere, making them accessible to plants^25,72^. Under in vitro conditions, rhizosphere bacteria, specifically Rb-18 and Rb-11, demonstrated the ability to solubilize inorganic phosphorus, potassium, and zinc. The solubilization of minerals was notably more efficient in rhizosphere bacteria compared to endophytic bacteria. Several studies have also demonstrated the involvement of rhizospheric *Bacillus* and *Pseudomonas* genera in the solubilization of phosphorus, potassium, and zinc under both controlled and field conditions, resulting in enhanced plant growth and yield^73–75^.

In the potted plant experiment, treatments T2 (*P*. *aeruginosa* + *F*. *udum*), T3 (*T*. *harzianum* + *F*. *udum*), T1 (*B*. *subtilis* + *F*. *udum*), and T4 (*T*. *asperellum* + *F*. *udum*) demonstrated a significant reduction in the incidence of wilt disease. This aligns with findings from previous studies^11,15,76^ which also found that native *Pseudomonas* spp., *Bacillus* spp., and *Trichoderma* spp. isolated from the rhizosphere of pigeonpea effectively reduced pigeonpea wilt disease under in vitro experiments.

Beneficial microorganisms often adopt an indirect strategy to enhance a plants resistance against invading phytopathogens by stimulating the plants defense mechanisms. In our study, we focused on inducing Systemic Resistance (ISR) in pigeonpea exposed to antagonistic microbes, including *B*. *subtilis*, *P*. *aeruginosa*, *T*. *harzianum*, *T*. *asperellum*, and *Trichoderma* sp., in the presence of the wilt causing pathogen *F*. *udum*. Additionally, we observed that plants inoculated with *F*. *udum* but lacking these bioagents exhibited a reduction in the activity of defense related antioxidant enzymes, including POD, PPO, and PAL. The increased activity of the host plant's defense system, particularly the enzymes POD, PPO, and PAL, can be attributed to the secretion of siderophores, chitinase, and protease by these microbes. These compounds act as signaling molecules that activate systemic resistance^21^. Several studies have demonstrated that Plant Growth Promoting Rhizobacteria (PGPR) can trigger various defense responses in host plant tissues, including the enhancement of antioxidant defense enzyme activity during pathogen attacks^77,78^. Multiple case studies provide evidence that the inoculation of PGPR can activate ISR related antioxidant enzymes, leading to a reduction in the severity of diseases caused by *F*. *udum* in pigeonpea. For instance, treatments involving *B*. *subtilis*, *P*. *aeruginosa*, and *Trichoderma* spp. have been shown to activate ISR related antioxidant enzymes, ultimately mitigating the impact of *F*. *udum* induced diseases in pigeonpea^7^.

In subsequent field investigations, the application of seed treatment with antagonistic microbes, including *P*. *aeruginosa* (33.33%), *T*. *harzianum* (35.41%), *B*. *subtilis* (36.66%), and *T*. *asperellum* (52.91%), demonstrated effectiveness in reducing the incidence of wilt disease in pigeonpea plants under disease challenged conditions. Numerous rhizosphere microbes have showcased their ability to alleviate the detrimental impacts of both biotic and abiotic stress factors, ultimately fostering plant growth and development^79^. Previous studies have indicated that *T*. *harzianum* and *T*. *asperellum* exhibit mycoparasitic activity against soil borne pathogens by releasing compounds such as stigmasterol and ergosterol^80,81^. Moreover, soil applications of *T*. *harzianum* have been demonstrated to reduce the population of *F*. *udum* in the soil, consequently decreasing the occurrence of pigeonpea wilt^15^. Additionally, *P*. *aeruginosa* produces antibiotics like oxychlororaphin and phenazine-1-carboxylic acid, which have proven effective in reducing *Fusarium* wilt in both chickpea and pigeonpea^82^. Extracellular proteins from *B*. *subtilis* have been found to induce flocculation and vacuolation in *F*. *udum* mycelium^76^. The diverse antimicrobial compounds produced by these beneficial microbes hinder the growth, metabolism, and pathogenicity of various fungal phytopathogens^52^. Consequently, these beneficial fungal and bacterial microbes effectively alleviate the severity of *F*. *udum* induced wilt disease. This observation is supported by a report from^83^ suggesting that antagonistic strains of *Pseudomonas*, *Bacillus*, and *Trichoderma* spp. genera, isolated from the pigeonpea rhizosphere, significantly reduce the severity of wilt disease caused by *F*. *udum* in host plants. Additionally, these rhizobacterial inoculations have been shown to enhance the growth characteristics of host plants compared to untreated controls^83^.

AMMI ANNOVA of all five Treatments (T) over four Environments (E) showed that 0.24% of the total SS was attributed to Environments (E) effect; 95% to Treatments (T) effects and 0.88% to Treatments by Environments (T x E) interaction effects. A large SS for Treatments (T) revealed the huge differences among the mean disease incidence causing most of the variations within the reactions of the treatments^84–86^.

## Conclusion

In summary, this study highlights the serious threat of *Fusarium* wilt in Pigeonpea and the limited effectiveness of conventional management methods. Indigenous biocontrol agents, such as *P*. *aeruginosa* (Eb-21), *T*. *harzianum*, and *B*. *subtilis* (Rb-18), have shown promise in controlling *Fusarium* wilt in both lab and field settings. They exhibited antagonistic activity against *F*. *udum*, boosted beneficial enzyme activity, and strengthened pigeonpea's resistance mechanisms. Over four seasons of field trials, treatments with *P*. *aeruginosa* and *T*. *harzianum* consistently had the lowest disease rates. This research emphasizes the potential of these biocontrol agents as sustainable alternatives to traditional fungicides and resistant cultivars for managing *Fusarium* wilt.

### Supplementary Information

Supplementary Information.

## Data Availability

The data presented in the study are deposited in the NCBI database (National Center for Biotechnology Information). Accession numbers submitted in NCBI: OR267399 (*Fusarium udum*), OR267401 (*Fusarium udum*), OR267402 (*Fusarium udum*), OR083610 (*Fusarium udum*), OR267395 *(Fusarium udum*), OR244422 (*Bacillus* sp*.)*, OR261238 (*B. subtilis*), OR244411 (*P. aeruginosa*), MZ348897 (*P. aeruginosa*), OR244366 (*Bacillus* sp), OR244404 (*B. subtilis*), OR244345 (*B. megaterium*), MZ348896 (*B. subtilis*), OR244371 (*B. velezensis*), MZ348898 (*T*. *harzianium*) MZ411690 (*T*. *asperellum*) and MZ411691 (*Trichoderma* sp.).
