# Supplementary material for: Harnessing the power of native biocontrol agents against wilt disease of Pigeonpea incited by Fusarium udum
Source: Sci Rep. 2024 May 31;14:12500. doi: 10.1038/s41598-024-60039-0 (PMC11143286; doi:10.1038/s41598-024-60039-0)
Supplement: Supplementary file 1 — Supplementary Information. [file 41598_2024_60039_MOESM1_ESM.docx]

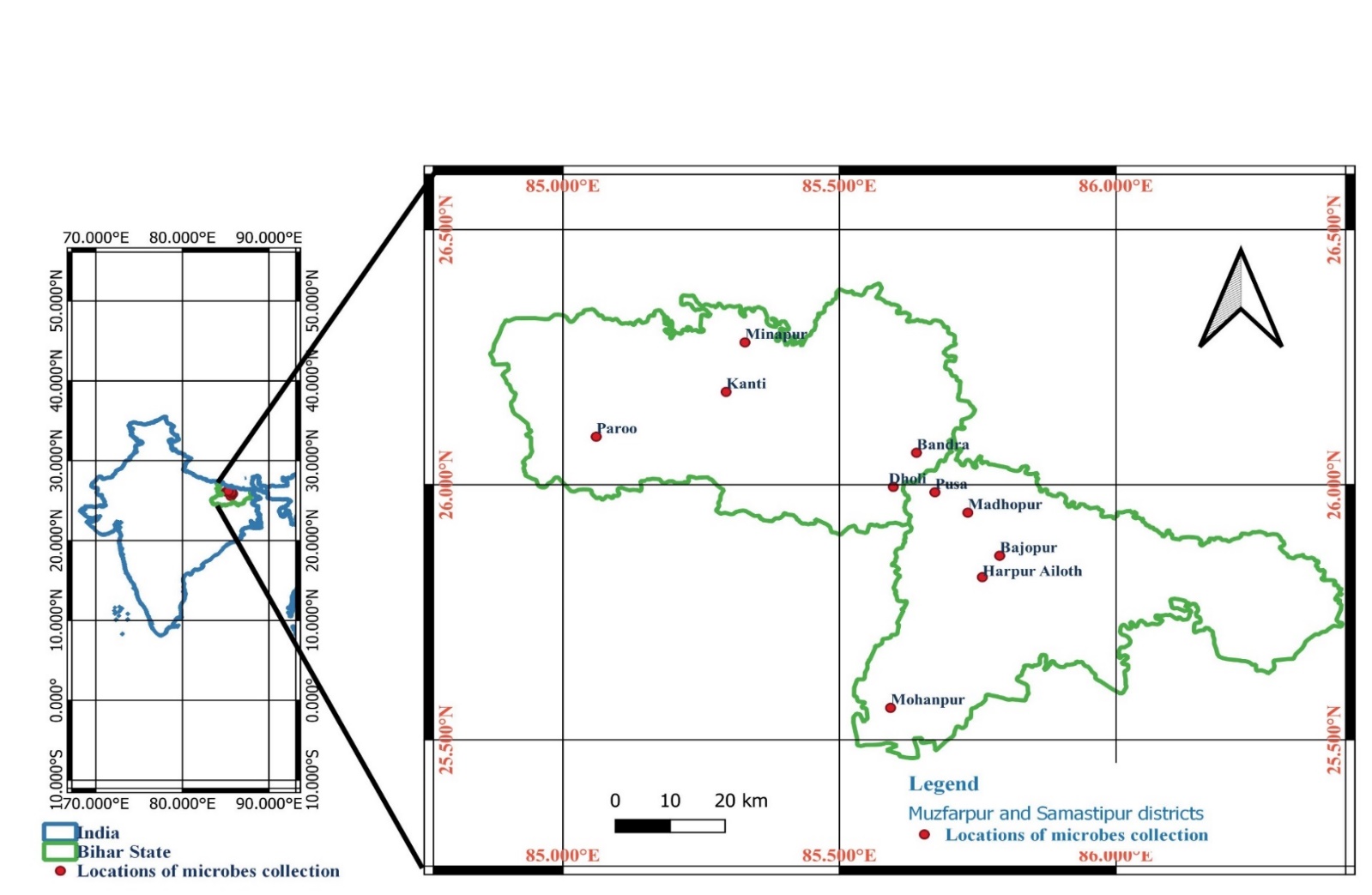


**Supplementary Figure 1.** Locations of soil and plant samples collection


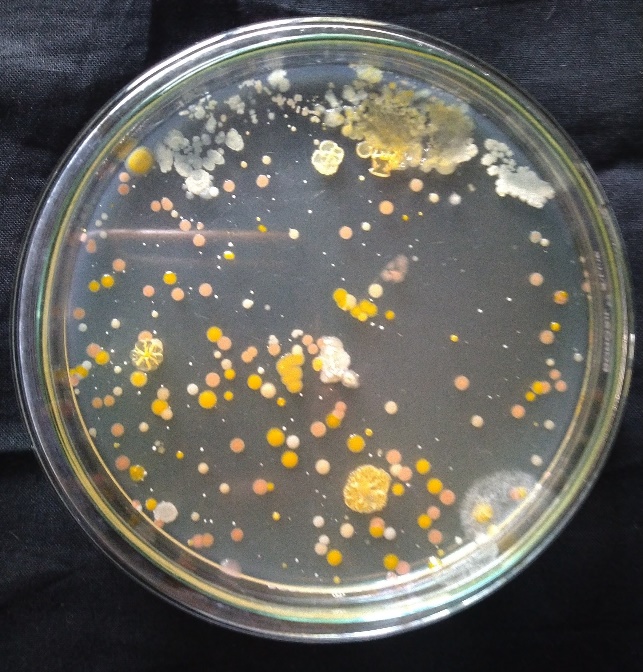

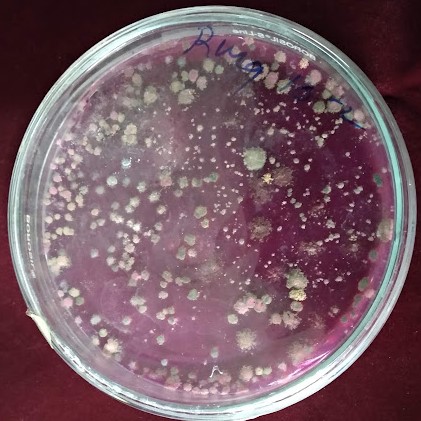


**A B**

**Supplementary Figure 2.** Serial dilutions for isolation of Biocontrol agents (A) Endophytic bacteria and (B) *Trichoderma*


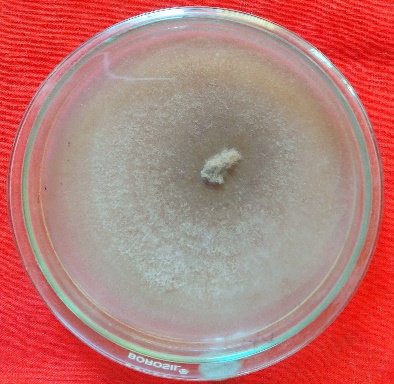

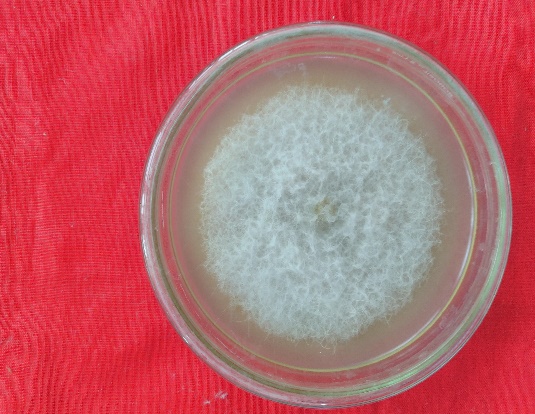

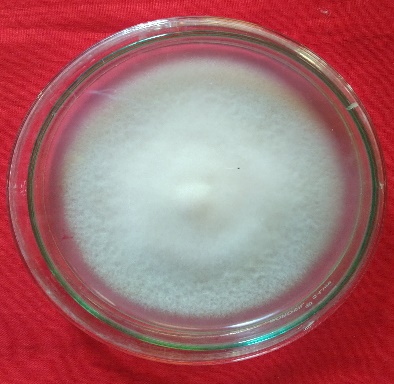

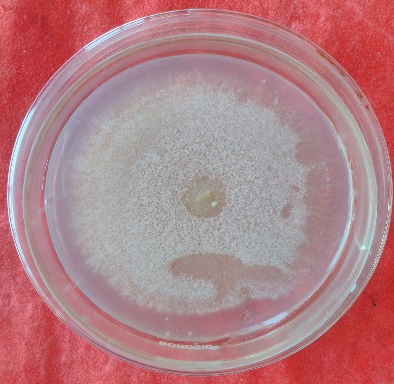


**ICP 2376**

**BAHAR**

**ICP 8862**

**ICP 8858**

**ICP 9174**


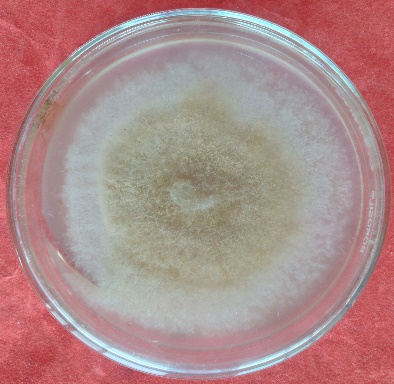


**Supplementary Figure 3.** *F. udum* isolates, isolated from various pigeonpea cultivars


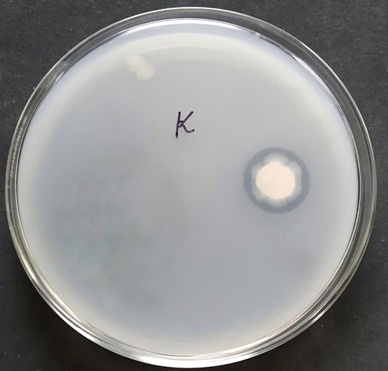


**Supplementary Fig 4.** Potassium solubilizing activity of bacterial isolates (Rb-18)

**Supplementary Figure 5.** Activity of Peroxidase (POD) in pigeonpea plants at different intervals of inoculation with biocontrol agents

**Supplementary Figure 6.** Activity of Polyphenol Oxidase (PPO) in pigeonpea plants at different intervals of inoculation with biocontrol agents

**Supplementary Figure 7.**  Activity of Phenylalanine Ammonia Lyase (PAL) in pigeonpea plants at different intervals of inoculation with biocontrol agents
